# Anti-glomerular basement membrane disease with intense nephrotic syndrome: a new case report

**DOI:** 10.11604/pamj.2021.39.243.30461

**Published:** 2021-08-16

**Authors:** Ikram Mami, Emna Ghzel, Rym Abida, Fadwa Hlaoui, Hela Jebali, Lamia Rais, Soumaya Beji, Fethi Ben Hamida, Lilia Ben Fatma, Karim Zouaghi

**Affiliations:** 1 Department of Nephrology, Dialysis and Kidney Transplantation, La Rabta Hospital, Tunis, Tunisia,; 2 Faculty of Medicine, University of Tunis El Manar, Tunis, Tunisia,; 3 Department B of Internal Medicine, Charles Nicolle Hospital, Tunis, Tunisia,; 4 Research Laboratory in Renal Pathology, Medicine School of Tunis, Tunis El Manar University, Tunis, Tunisia

**Keywords:** Anti-glomerular, basement membrane disease, nephrotic syndrome, proteinuria, case report

## Abstract

Anti-glomerular basement membrane (anti-GBM) disease was usually described as a small vessel vasculitis presenting with acute kidney injury, haematuria and non-nephrotic proteinuria. We report a case of anti-GBM disease revealed by an intense nephrotic syndrome. The urinary protein level was 12g/day. Renal biopsy only showed crescent glomerulonephritis with linear staining of IgG in direct immunofluorescence without other glomerulonephritis. Immunoglobulin G (IgG) anti-GBM antibody titer was elevated.

## Introduction

Anti-glomerular basement membrane (anti-GBM) disease is a rare condition causing renal and/or alveolar damage [1]. It is an auto-immune condition triggered by immune complexes formed by immunoglobulin G (IgG) auto-antibodies against the alpha 3 chain of type IV collagen [1]. Renal manifestations of this disease typically include hematuria, non-nephrotic proteinuria and rapidly progressive renal failure [1]. Clinical presentation of anti-GBM disease by nephrotic syndrome is rare. We hereby present a case of anti-GBM disease revealed by an intense nephrotic syndrome.

## Patient and observation

**Patient information:** a 25-year-old male patient, smoker, without previous exposure to toxic substances, coming from a rural area, was admitted to the nephrology department for a nephrotic syndrome that was discovered after a pulmonary infection.

**Clinical findings:** clinical examination found a general state without fever. Edema was important. Arterial pressure was 160/90mmHg. Urine dipstick analysis showed positive hematuria and 3 marks of proteinuria. Pulmonary examination was normal and so was the rest of the clinical assessment.

**Figure 1:**
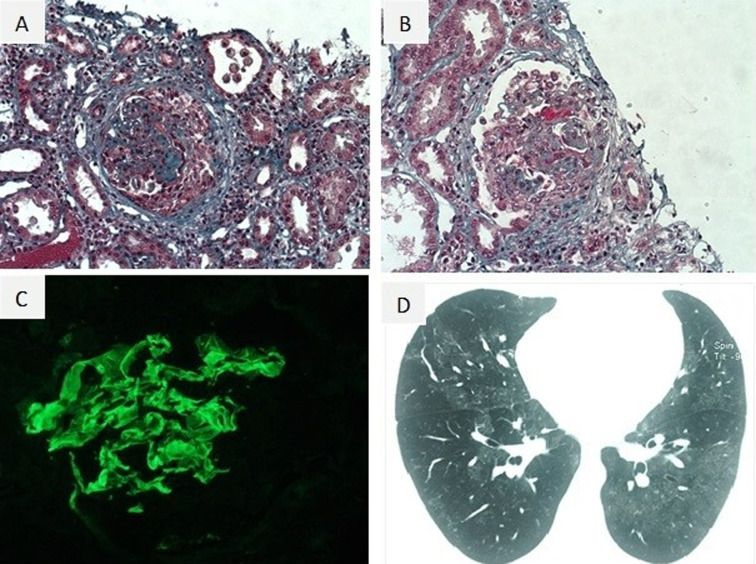
A) renal biopsy, Masson’s trichrome (x200), circumferential cellular crescent; B) renal biopsy, Masson’s trichrome (x200), capillary fibrinoid necrosis; C) renal biopsy, immunofluorescence (x200); linear anti-IgG antibody staining along the GBM; D) computed tomography showed diffuse alveolar hemorrhage

**Diagnostic assessment:** laboratory tests revealed a renal failure with a creatinine level at 274μmol/l, an intense nephrotic syndrome with serum protein level at 38g/l, serum albumin level at 18g/l and urinary protein level of 12g/24 hours, as well as a microcytic anemia at 8.7g/dl. Chest radiography was normal. Renal ultrasonography found kidney measurements within normal range with preserved corticomedullary differentiation and contours. Considering all the information above, our patient had an impure nephrotic syndrome with an anemia inadequately proportional to the level of the renal failure. Perinuclea anti-neutrophil cytoplamic (p ANCA), cytoplasmic anti-neutrophil cytoplasmic (c ANCA) antibodies and antinuclear antibodies (AAN) were negative. Serum complement is normal. However, an Immunoglobulin (IgG) anti-GBM antibody titer was elevated. Renal pathology of 25 glomerulus found large cellular crescents in 17 glomeruli: circumferential in 11 glomeruli and circumscribed in 7 glomeruli, without rupture of Bowman´s membrane (Figure 1). There were also segmental sclerotic lesions with flocculo-capsular apposition in 2 glomeruli alongside with extensive tubular necrosis lesions. Linear staining of IgG were found in direct immunofluorescence. Moreover, anti-GBM antibodies were positive. Diagnosis of anti-GBM disease was confirmed. Computed Tomography showed alveolar hemorrhage.

**Therapeutic intervention:** treatment was immediately initiated: one gram per day of intravenous bolus methylprednisolone for 3 days relayed by oral prednisone intake at a dosage of 1mg/kg/d, associated with plasmapheresis as 6 intravenous boli of 500mg of cyclophosphamide: every 2 weeks for a month then every 3 weeks.

**Follow-up and outcomes:** pulmonary outcome was favorable. Anti-GBM antibodies were negative after 12 sessions of plasmapheresis. However, renal function had dramatically decreased leading to a terminal chronic kidney disease and periodic hemodialysis within one month.

## Discussion

Anti-GBM disease is a small vessel vasculitis involving capillaries of the kidneys and the lungs. It classically characterized by rapidly progressive glomerulonephritis, associated or not with intra-alveolar hemorrhage (Goodpasture syndrome) [2]. The disease has a bimodal distribution as it is predominately noted within young males and older females. Smoking seems to be the main risk factor [3]. Our observation is distinctive by the existence of an intensive nephrotic syndrome with extensive proteinuria ranging at 12g/24 hours. Such nephrotic syndrome had rarely been described in the course of good pasture disease. Few cases were reported in the literature with documented nephrotic range proteinuria [4-7] (Table 1). Moreover, larger studies had confirmed the low frequency of nephrotic syndrome in goodpasture [8,9]. The various studies sought to find an explanation for this association between the GoodPasture syndrome and nephrotic syndrome. It has been previously described that anti-GBM disease with nephrotic syndrom can be associated to others glomerulonephritis such as membranous nephropathy (MN) and minimal change disease [10]. However, simultaneous anti-GBM disease and MN were the association the most described in the literature [10]. Association of anti-GBM glomerulonephritis and MN was explained by of immune complex deposits in the sub-epithelial space [8]. In our presentation, nephrotic syndrome could not be explained by renal biopsy results subject to an electron microscopy study. Standard treatment for anti-GBM disease is aggressive, including plasmapheresis along with cyclophosphamide and corticosteroids [2]. Zhong *et al*. also reported a case report of anti-GBM disease with nephrotic syndrome treated by Tacrolimus with partial remission [6]. Renal progression was unfavourable and the diagnosis of the terminal stage was retained with initiation of hemodialysis after one month.

**Table 1:** anti-glomerular basement membrane disease with nephrotic syndrome: summary of literature

Case report	Age (years)	Gender	Nephrotic range proteinuria (g/day)	Serum creatinine (mg/l)	Serological anti-GBM antibody	Renal biopsy	Outcomes
Qunibi *et al*. (1979) 1^st^ case 2nd Case	51	M	7.5	48	positive	Crescentic glomerulonephritis IF : Ig G	Hemodialysis
	37	M	7,3	18	negative	Crescentic glomerulonephritis IF : Ig G	Stable renal function
Okafor *et al*. (2011)	60	F	22.5	24	positive	Crescentic glomerulonephritis IF : Ig G	Hemodialysis
Zhong *et al*. (2020)	38	M	7.4	8	negative	Crescentic glomerulonephritis IF : Ig G, C3	Stable renal function partial remission of nephroti syndrome
Yuko Shibata *et al*. (2020)	30	M	5.28	66	positive	Crescentic glomerulonephritis IF : Ig G	
Present case	25	M	12	31	positive	Crescentic glomerulonephritis IF : Ig G	HD

## Conclusion

Goodpasture syndrome is to be called to mind, even in cases of heavy proteinuria. This highlights the important role of renal biopsy in the differential diagnosis of such unusual clinical presentations and the importance of histological study of the electron microscope to reveal podocytopathies associated with Goodpasture syndrome.
